# Therapist-Guided Tablet-Based Telerehabilitation for Patients With Aphasia: Proof-of-Concept and Usability Study

**DOI:** 10.2196/13163

**Published:** 2019-04-26

**Authors:** Stephan Moreno Gerber, Narayan Schütz, Arif Sinan Uslu, Nadine Schmidt, Carina Röthlisberger, Patric Wyss, Sandra Perny, Corina Wyss, Monica Koenig-Bruhin, Prabitha Urwyler, Thomas Nyffeler, Laura Marchal-Crespo, Urs Peter Mosimann, René Martin Müri, Tobias Nef

**Affiliations:** 1 Gerontechnology & Rehabilitation Group University of Bern Bern Switzerland; 2 Department of Neurology, University Neurorehabilitation University Hospital Bern (Inselspital) University of Bern Bern Switzerland; 3 Neurocenter Luzerner Kantonsspital Luzern Switzerland; 4 ARTORG Center for Biomedical Engineering Research University of Bern Bern Switzerland

**Keywords:** aphasia, high-intensity training, telerehabilitation, multiplatform system

## Abstract

**Background:**

Aphasia is the loss or impairment of language functions and affects everyday social life. The disorder leads to the inability to understand and be understood in both written and verbal communication and affects the linguistic modalities of auditory comprehension, verbal expression, reading, and writing. Due to heterogeneity of the impairment, therapy must be adapted individually and dynamically to patient needs. An important factor for successful aphasia therapy is dose and intensity of therapy. Tablet computer–based apps are a promising treatment method that allows patients to train independently at home, is well accepted, and is known to be beneficial for patients. In addition, it has been shown to ease the burden of therapists.

**Objective:**

The aim of this project was to develop an adaptive multimodal system that enables aphasic patients to train at home using language-related tasks autonomously, allows therapists to remotely assign individualized tasks in an easy and time-efficient manner, and tracks the patient’s progress as well as creation of new individual exercises.

**Methods:**

The system consists of two main parts: (1) the patient’s interface, which allows the patient to exercise, and (2) the therapist’s interface, which allows the therapist to assign new exercises to the patient and supervise the patient’s progress. The pool of exercises is based on a hierarchical language structure. Using questionnaires, therapists and patients evaluated the system in terms of usability (ie, System Usability Scale) and motivation (ie, adapted Intrinsic Motivation Inventory).

**Results:**

A total of 11 speech and language therapists (age: mean 28, SD 7 years) and 15 patients (age: mean 53, SD 10 years) diagnosed with aphasia participated in this study. Patients rated the Bern Aphasia App in terms of usability (scale 0-100) as excellent (score >70; *Z*=–1.90; *P*=.03) and therapists rated the app as good (score >85; *Z*=–1.75; *P*=.04). Furthermore, patients enjoyed (scale 0-6) solving the exercises (score>3; mean 3.5, SD 0.40; *Z*=–1.66; *P*=.049).

**Conclusions:**

Based on the questionnaire scores, the system is well accepted and simple to use for patients and therapists. Furthermore, the new tablet computer–based app and the hierarchical language exercise structure allow patients with different types of aphasia to train with different doses and intensities independently at home. Thus, the novel system has potential for treatment of patients with aphasia as a supplement to face-to-face therapy.

## Introduction

Language and speech are crucial to communication and play a central role in everyday social life. Aphasia is an acquired language impairment that follows brain injury. It affects the linguistic modalities of auditory comprehension, verbal expression, reading, and writing and must be distinguished from other cognitive communication problems [1]. Aphasia is common in patients with stroke and traumatic brain injury. In acute stroke, it affects about one-third of the patients [2,3]. The recovery depends mainly on the type of aphasia and severity of the initial insult. The recovery rate is highest during the first 3 months [4]. Patients with aphasia not only differ in the degree of language impairment but also in cognitive functioning and communication capabilities [5].

The most common treatment of aphasia is direct retraining of the linguistic deficits. Alternative therapy forms include pharmaceutical drugs or treatment of neurobehavioral functions [6]. A study conducted by Grechuta et al suggested that silent visual cues facilitate word retrieval and verbal execution and thus improve language functions [7]. Another effective strategy to improve word retrieval and auditory comprehension is intensive language-action therapy, combining speaking and writing with nonlinguistic actions [8,9].

However, there is a lack of consensus between the relationship of dose and intensity of the therapy [10]. Dignam et al showed that distributed therapy over 8 weeks showed higher improvement in language functions than intense therapy over 3 weeks [11], but there is evidence that intensive face-to-face therapy time improves later outcome [12-15]. Furthermore, therapy should commence as early as possible after stroke incidence [16].

The feasibility of intense face-to-face therapy in clinics is limited, since it requires a sufficient number of qualified therapists and is quite expensive. Therefore, the advent of computer-aided therapy is increasing, since it reduces the load of therapists while maintaining and augmenting established therapy [17]. Evidence has shown that computer-based training, in addition to established therapy, is feasible and improves later outcomes [18-20]. For example, Lee et al developed a computer-based system where patients have to mimic the observed action to improve speech after stroke [21].

In particular, the use of tablet computers with touch screen manipulation has opened new opportunities for therapeutic purposes. Compared to paper-pencil exercises, they are more intuitive to use and highly portable [17]. Moreover, telerehabilitation apps allow therapists to treat and provide remote support and feedback through telerehabilitation technology.

The state-of-the-art telerehabilitation apps for aphasic patients mostly focus on disorder-oriented treatment to restore the linguistic processing ability, whereas fewer apps focus on functional treatment to develop strategies to compensate for the deficit [22,23]. Evidence has shown that remotely delivered computer aphasia training is acceptable [24-27] and beneficial for patients [28-33].

To be practical, telerehabilitation apps should focus on multiple linguistic modalities. There are several validated systems on the market (eg, Constant Therapy, Tactus Therapy, and StepByStep Aphasia Therapy), which have shown evidence that tablet computer-based aphasia therapy focusing on multiple linguistic modalities is beneficial and improves later outcome [18,26,34].

Due to the heterogeneity of the aphasic population, the speech and language therapy, and thus intensity and dose, must be adapted dynamically to the individual patient’s needs. Until now, speech and language therapists had to create exercises based on recommendations and heuristics, which is very time consuming, as the therapists must develop a number of tasks to supplement the patients with enough training materials to avoid repetition.

Additionally, the ageing demographic and increase in the number of aphasic patients create a significant need for new adaptive multimodal telerehabilitation methods to improve the later outcome, quality of life, and probability of returning to work.

Therefore, in this study, we propose a new adaptive multimodal telerehabilitation system for patients with aphasia, which allows control of the dose and frequency of speech and language therapy remotely. We hypothesize that the developed system is simple and intuitive to use for both therapists and patients. This includes the ability for therapists to assign new exercises easily and efficiently based on an adaptive hierarchical language exercise structure, create new exercises for all linguistic modalities, give progress feedback to the patient, and access the system from different locations.

## Methods

### General Concept of the Bern Aphasia App

**Figure 1:**
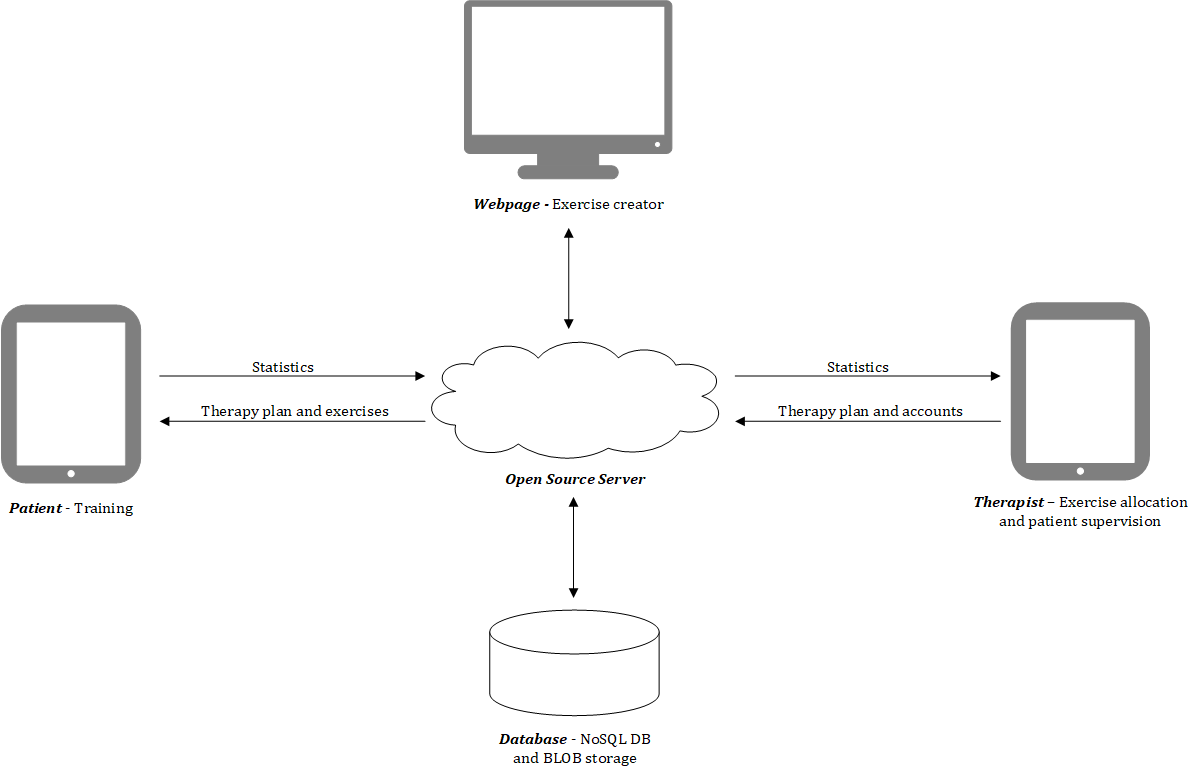
The general concept of the Bern Aphasia App with the five main parts and related data flow (ie, webpage to create exercises, NoSQL database and BLOB storage to store patient and exercise data, tablet computer interfaces [patient and therapist, iPad], and the open source server as backend service). NoSQL: Not Only Structured Query Language; DB: database.

The system was developed by the University of Bern along with the speech and language therapist of the University Hospital of Bern (Inselspital). The system contains five main parts (Figure 1): (1) The patient interface that allows the patient to train according to the therapy plan. (2) The therapist interface that allows the therapist to assign new exercises to the patient, create new patient accounts, and supervise the progress of the patient. The patient and therapist interfaces run on tablet computers (iPad) programmed in Object-C. (3) The Web page to create new exercises independent of the app. (4) The NoSQL (Not Only Structured Query Language) database to store patient and exercise data, and the Binary Large Objects storage to store all images and videos of the exercises. The database is located behind a secure firewall on a managed server and can only be accessed by authorized personnel (ie, administrator of the system). (5) The open-source server served as a backend service.

### Multimodal Exercises

In collaboration with speech and language therapists, 10 different exercise types were defined to meet all linguistic modalities (Table 1). More than 30,000 exercises in German were implemented, and new exercises can be added online by the exercise creator. The exercises are split into training units (deck), consisting of about 25 tasks.

**Figure 2:**
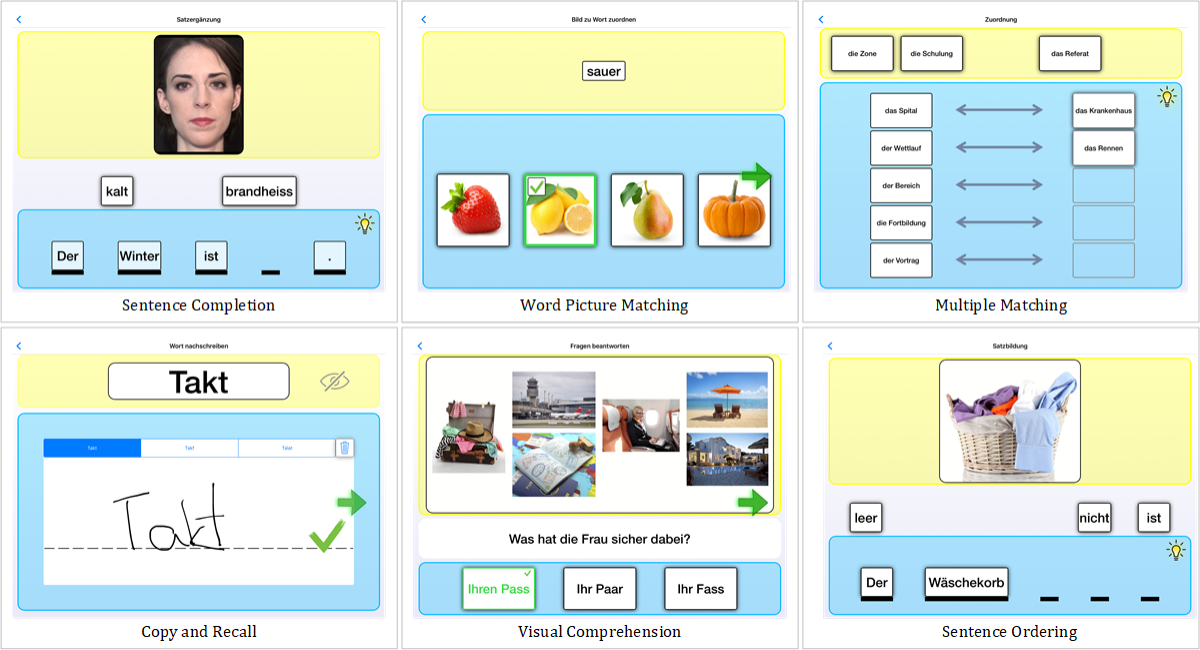
Set of possible exercise types and tasks.

The exercises consist of three main elements (ie, fixed, response, and supportive elements). Fixed elements build the structure of exercises and cannot be moved. Response elements are needed to complete the task and can be moved to fulfill the task. The number of fixed and response elements can be varied and are either videos, images, written language, placeholders, or audio tracks. Supportive elements (ie, videos, images, and audio tracks) act as an aid to solve the tasks and can thus be used to fine tune task difficulty. For example, in the sentence completion task in Figure 2, “kalt” (cold) and “brandheiss” (boiling) are response elements, whereas “Der” (The), “Winter” (winter), “ist” (is), and “.” are fixed elements. The video of a speech and language therapist spelling the correct word acts as a supportive element.

Based on the broad spectrum of impaired language functions, the difficulty of an exercise differs in patients, and thus, categorization into one difficulty measure is not possible and must be adapted individually. Therefore, a hierarchical system that is structured according to characteristics (ie, based on linguist rules) and difficulty within an exercise type (eg, phonology, morphology, syntax, semantics, and pragmatics) was implemented. Detailed information of each exercise type and the hierarchical structure can be found in the Multimedia Appendices 1 and 2.

### Workflow

**Figure 3:**
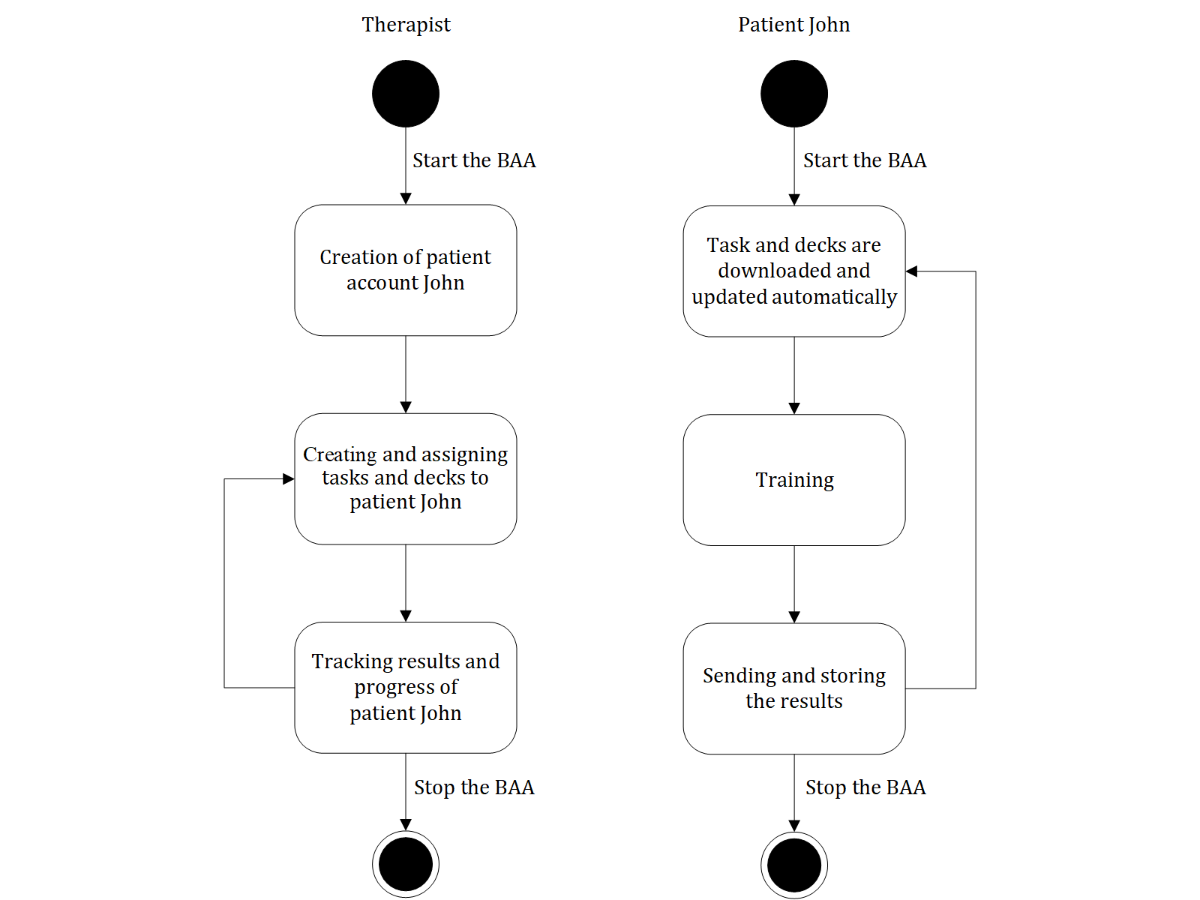
The general workflow of the Bern Aphasia App (BAA).

The therapist has to first create an account for the patient. Depending on the type and severity of the language impairment, the therapist assigns tasks and the corresponding decks to the patient. The tasks and decks are downloaded automatically onto the patient’s tablet computer. Each time the patient trains using the tablet computer, results are sent to the patient’s database. Feedback about the correctness of response is given automatically after the exercise is completed. In parallel, the therapist is able to change the exercise types and decks remotely, according to the needs of the patient. Furthermore, the therapist can monitor the patient’s progress in real time. The workflow is presented in Figure 3.

**Table 1:** Exercise categories.

Category and level		Exercise type		First level of the hierarchical language structure	Description	Supportive Media	
**Assigning**							
	Phonology		Single picture-word matching	Adjective, substantive, verb	Selecting the correct word from phonematically respective semantically related distractors		Audio, video
	Phonology		Single word-picture matching	Adjective, substantive, verb	Selecting the correct picture from phonematically respective semantically related distractors		Audio, video
	Semantic		Multiple Matching	Homonym, antonym	Matching all objects (word-picture, picture-picture, word-word)		—^a^
**Insertion**							
	Phonology		Word completion	Number of phonemes	Selecting the correct letter(s) (from distractors) and inserting them into the correct position(s)		Audio, image, video
	Phonology		Sentence completion	Grammatical syntactic and semantic processing	Selecting the correct words(s) (from phonematically respective semantically related distractors) and inserting them into the correct position(s)		Audio, image, video
**Sort**							
	Phonology		Anagram	Phonematic and semantic criteria	Bringing the letters into the correct order		Audio, image, video
	Grammar		Sentence ordering	3-6 words	Bringing the words into the correct order		Audio, image, video
**Mimic**							
	Phonology		Word repetition	Number of syllables, hierarchic, mixed, consonant clusters	Repeating the audio-visually recorded spoken word by a speech and language therapist		—
**Writing**							
	Phonology		Copy and recall	Low- and high- frequency words	Copying and recalling presented words by typing or writing		—
**Comprehension**							
	Auditory		—	—	Selecting the correct answer to a question about the auditory-based information		—
	Audio-visual		—	—	Selecting the correct answer to a question about the audio-visual based information		—
	Reading		—	Narrative and procedural	Selecting the correct answer to a question about the text-based information		—
	Visual		—	—	Selecting the correct answer to a question about the image-based information		—

^a^Not available.

**Adapted intrinsic motivation inventory questionnaire scale showing the subscales and items within each subscale.**

- **Feasibility:**
  - The training sequences were too long.
  - The training was too difficult for me.
- **Interest/Enjoyment:**
  - This activity was fun to do.
  - I thought the training was boring.
  - I thought the training was frustrating.
  - I liked to exercise.
  - I thought the training was arduous.
  - I thought the training was enjoyable.
  - I thought the training was very interesting.
- **Value/Usefulness:**
  - I thought the training helped me to feel better.
  - I would like to continue the training in the future.
- **Pressure/Tension:**
  - I felt pressured during the training.
  - I felt very tense while doing this activity.
  - I was worried about getting the training right.

### Procedure, Subject Recruitment, and Demographics

The study was divided into two phases: the development phase followed by evaluation of the system by patients and therapists. The study was carried out in accordance with the current version of the Declaration of Helsinki and approved by the Ethics Committee of the Canton of Bern, Switzerland. The participants (patients and therapists) were recruited via the University Hospital of Bern. Prior to participation, written informed consent was obtained, and procedures related to the study were explained to the participants. The main inclusion criteria for patients were age >18 years, diagnosis of aphasia, and a minimal level of cognitive function to handle a tablet computer and to understand the task and questionnaire. Patients with hemiparesis were not excluded from the study. For therapists, neither inclusion nor exclusion criteria were set. The study was conducted in the general ward (ie, neurorehabilitation) at the University Hospital Bern, and questionnaires were completed during the patient’s stay.

### Evaluation of the System

To assess patients’ and therapists’ opinion, attitude, and perception of the system, the well-established usability scale (System Usability Scale) [35] was used. The System Usability Scale is based on 10 questions and has a 5-point scale (1=strongly disagree, 5=strongly agree). To measure the patient’s subjective experience, enjoyment, and stress experienced, an adapted selection of 14 items of the intrinsic motivation inventory questionnaire was used (Textbox 1) [36]. The adapted Intrinsic Motivation Inventory questionnaire encompassed the dimensions of Feasibility, Interest/Enjoyment, Value/Usefulness, and Tension/Pressure based on a 7-point scale (0=strongly disagree, 6=strongly agree). For patients’ understanding, the questionnaires were explained and filled out with the therapists. The assignment of exercises based on diagnostic tests and the assessment of the system was performed by the same therapist. The intrinsic motivation inventory questionnaire was added later to the study and was therefore not assessed in all patients.

### Statistical Analysis

Analysis was conducted using R (R Foundation for Statistical Computing, Vienna, Austria), whereas for the intrinsic motivation inventory, a nonparametric Wilcoxon signed rank test was used, which accounts for a small sample size. For the subscale Interest/Enjoyment, Value/Usefulness, and Feasibility, a score>3 (ie, mean of the score scale) and for Pressure/Tension, a score<3 was regarded as positive and significant. To analyze the System Usability Scale, a nonparametric Wilcoxon signed rank test was used if the score was significantly above 70 (Good) or 85 (Excellent) [37].

## Results

Overall, the Bern Aphasia App was used by 166 patients who solved 82,891 cards (64,144 cards solved correctly) and exercised for a mean of 3.96 (SD 21.88) hours. Furthermore, while in use (required internet connection), the Bern Aphasia App ran stably without any technical issues.

**Figure 4:**
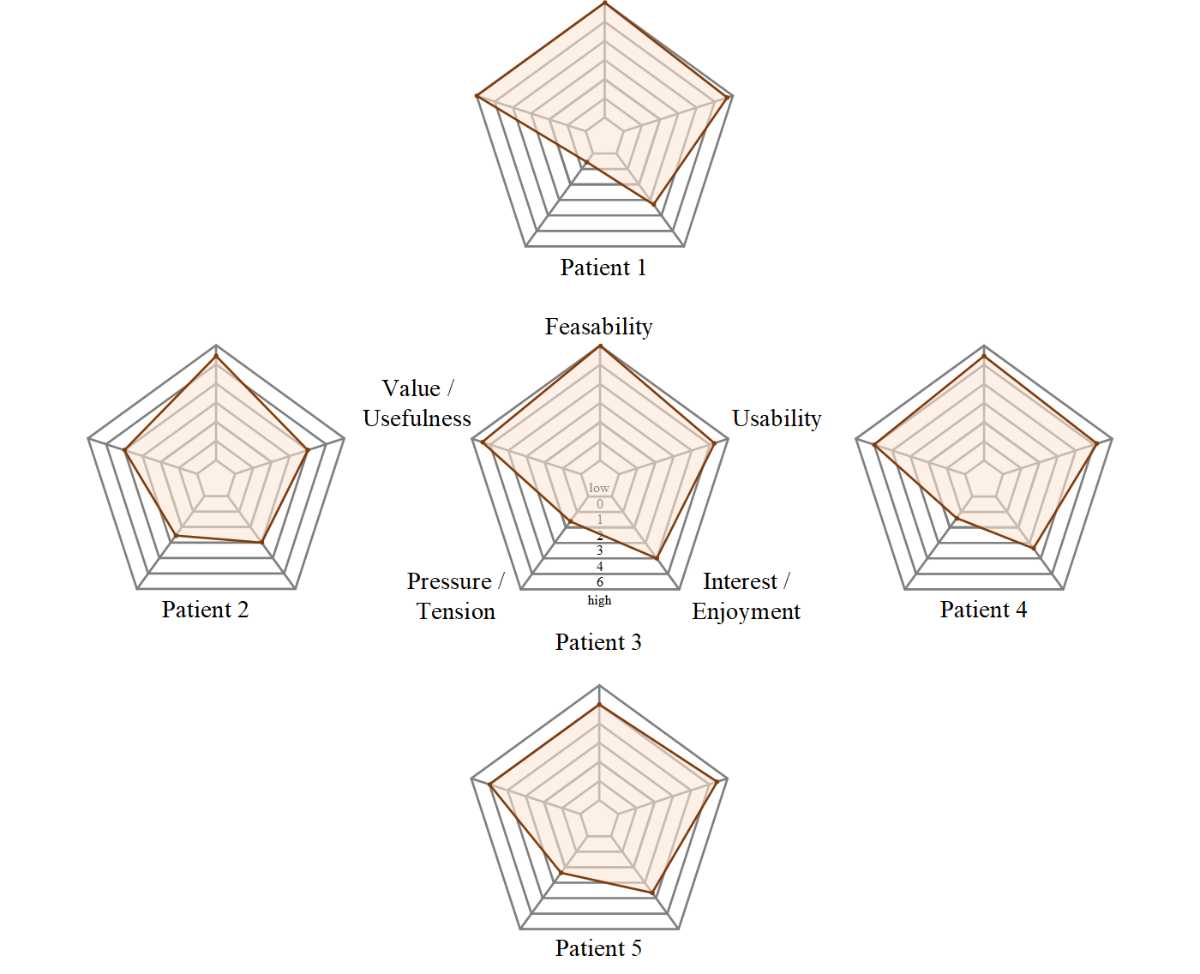
Patients’ answers to the intrinsic motivation inventory questionnaire (ie, Interest/Enjoyment, Pressure/Tension, Value/Usefulness, and Feasibility) and the System Usability Scale (ie, Usability).

A total of 11 (10 female and one male) experienced speech and language therapists (age: mean 28, SD 7 years), and 15 of all patients using the Bern Aphasia App (12 male and 3 female patients) diagnosed with aphasia (age: mean 53, SD 10 years) participated for an average of 444 days after the incident (SD 427.61 days) in the study. Based on the therapist’s diagnosis, patients had moderate to severe aphasia due to stroke or traumatic brain injury.

The usability, scored between 0 and 100, revealed that both patients (mean 90.0, SD 8.9) and therapists (mean 75.5, SD 8.2) scored the Bern Aphasia App above the mean of the score scale. Patients rated the Bern Aphasia App in terms of usability as excellent (Z=–1.90, *P*=.03) and therapists rated the app as good (Z=–1.75, *P*=.04).

Five of the 15 patients also filled out the intrinsic motivation inventory questionnaire (Figure 4). On an average, the Feasibility (mean 5.6, SD 0.42; *Z*=–0.91; *P*=.03), Interest/Enjoyment (mean 3.5, SD 0.40; *Z*=–1.66; *P*=.049), and Value/Usefulness (mean 5.1, SD 0.74; *Z*=–1.90; *P*=.03) of the training were rated significantly higher than the mean of the score scale (range of the score scale =0-6), whereas Pressure/Tension was rated significantly lower, close to the minimum of the score scale (mean 1.7, SD 0.80, *Z*=–1.86, *P*=.03; Figure 2).

## Discussion

### Principal Results

In this study, we developed a multimodal telerehabilitation system to train the linguistic modalities in patients with aphasia. In line with our hypothesis, the system is simple to use, highly adaptable to the patient’s need, and highly accepted. It ran stably and was appreciated by patients as well as therapists.

### Patient Interface

The first main finding is that the questionnaire response in terms of usability was rated as excellent, and thus, the developed system is well accepted by patients.

Training with the Bern Aphasia App was rated as enjoyable, which confirms that the personalized content and difficulty of exercises could be adapted by therapists to the needs of patients. The slight increased pressure and tension to train indicate that the exercises were challenging but not frustrating. Overall, the usability and motivation indicate that the design for the patient interface is clear and consistent and thus offers the possibility of training independently. The intrinsic motivation score was consistent with that in the literature, whereas the usability score was higher [38,39].

### Therapist Interface

The second main finding was that the new system allows therapists to adapt and monitor the training of the patient remotely. The usability for the therapist interface was rated lower than that for the patients’ interface but still considered good. One reason for the lower usability score might be the need for more functionality in the therapist interface compared to the patient interface and thus the need for more time for familiarization. Another reason for the lower usability score in the therapist interface could be that the patient interface was better designed and thus more adapted to their needs.

### Limitations and Outlook

Due to the study design of a feasibility study, it remains unclear whether this result can be generalized to all patients with aphasia and therapists and whether evaluation can be transferred from a clinical to a home setting. An additional limitation of this study is the small sample size of patients and therapists and that the therapist who assigned exercises and conducted the study was the same person.

Furthermore, when using the app, patients must be connected to the internet. In rare cases, the internet connection was too slow, or patients lost internet connection and had trouble reconnecting. Therefore, it is crucial that future telerehabilitation apps can be used offline while exercising and that internet connection is only needed to synchronize the app (ie, loading new exercises and sending statistical reports to the therapist).

In the next step, we will investigate the effect of the Bern Aphasia App in a randomized multicenter clinical trial at patients’ homes. Positive results in clinical trials could have a great socioeconomic impact in addition to increased quality of life of the affected patients. With tablet-based apps like the Bern Aphasia App, both patients and therapists can benefit from an intuitive, cost-efficient, touch-based reliable product that fits well with the current trend of moving health treatment from hospital to home. We suggest standard linguistic tests (eg, Boston naming [40], Token test [41], and Amsterdam-Nijmegen everyday language [42]) prior to and after the intervention as a primary outcome and follow-up measurement as well as questionnaires about motivation and quality of life as secondary outcomes to determine the actual improvement caused by the therapy exercises.

The exercises in the hierarchical language structure are usually created by recommendations and heuristics, which is highly time consuming. Therefore, future research should focus on automated exercise creation based on artificial intelligent algorithms to ease the burden of therapists.

### Conclusions

Based on the questionnaire scores, the developed system is well accepted and simple to use for patients and therapists. The tablet computer–based app and the hierarchical language exercise structure offer patients with different forms of aphasia a possible chance to train with different doses and intensities independently at home. Overall, the novel system has potential for treatment of patients with aphasia as a supplement to face-to-face therapy.

## Appendix

Multimedia Appendix 1 Exercises in the Bern Aphasia App.

Multimedia Appendix 2 Bern Aphasia App video.

## Abbreviations

NoSQL: Not Only Structured Query Language
