# Supplementary material for: Therapist-Guided Tablet-Based Telerehabilitation for Patients With Aphasia: Proof-of-Concept and Usability Study
Source: JMIR Rehabil Assist Technol. 2019 Apr 26;6(1):e13163. doi: 10.2196/13163 (PMC6658255; doi:10.2196/13163)
Supplement: Multimedia Appendix 1 [file rehab_v6i1e13163_app1.pdf]

Exercises Bern Aphasia App

1 Category Assigning 2
1.1 Single picture-word matching 2
1.2 Single word-picture matching 3
1.3 Multiple matching 4
2 Category Insertion 5
2.1 Word completion 5
2.2 Sentence completion 6
3 Category Sort 7
3.1 Anagram 7
3.2 Sentence ordering 8
4 Category Mimic 9
4.1 Word repetition 9
5 Category Writing 10
5.1 Copy and recall 10
6 Category Comprehension 11

1 Category Assigning

1.1 Single picture-word matching

The task of single picture-word matching is to select the correct word from phonematically respective semantically related distractors (i.e. words). For example (figure 1), which of the words matches the image of an **ancient document** best? Old would be the correct answer, whereas new, digital, electronic, and visual would be the distractors. The number of distractors can be varied. Additionally, as supportive elements to the picture, a video or an audio file of a speech and language therapist spelling the correct word can be displayed. To adapt the task to the patients, the exercise structure (figure 2) is hierarchically structured into decks focused on adjectives, substantives, and verbs.

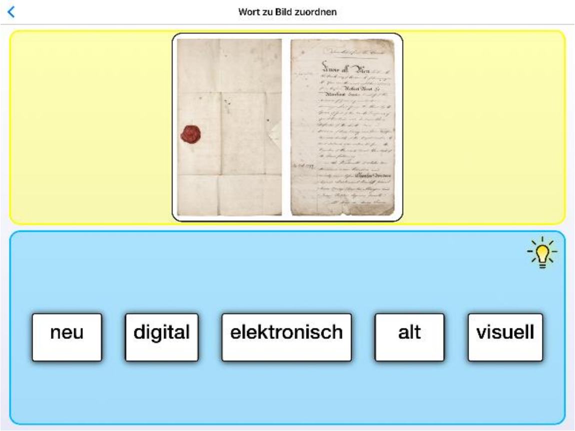

Figure 1: Exercise single picture-word matching

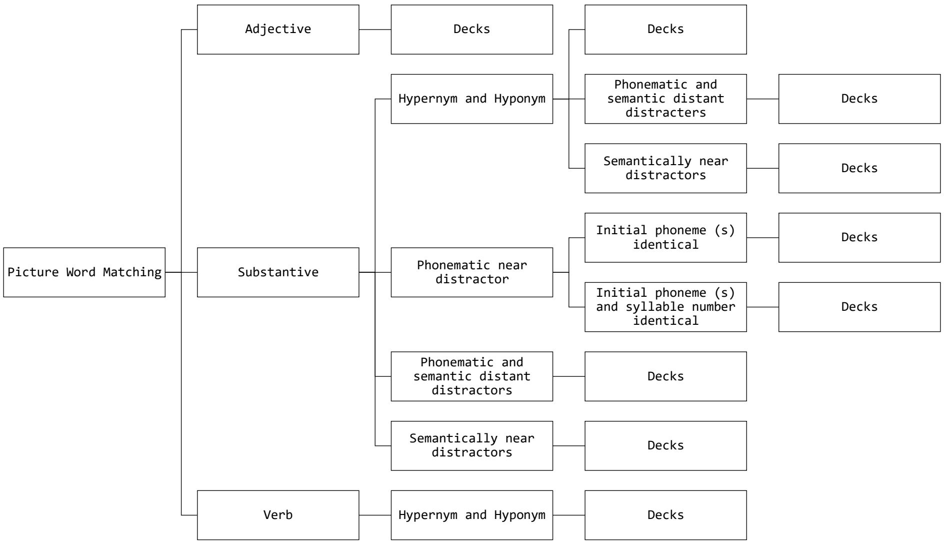

Figure 2: Exercise structure single picture-word matching

1.2 *Single word-picture matching*

The task of single word-picture matching is to select the correct picture from phonematically respective semantically related distractors (i.e. pictures). For example (figure 3), which of the images contains a **sour** fruit/vegetable? Lemon would be the correct answer, whereas strawberry, pear, and pumpkin would be the distractors. The number of distractors can be varied. Additionally, as supportive elements to the word, a video or audio file of a speech and language therapist spelling the correct word can be displayed. To adapt the task to the patients, the exercise structure (figure 4) is hierarchically structured into decks focused on adjectives, substantives, and verbs.

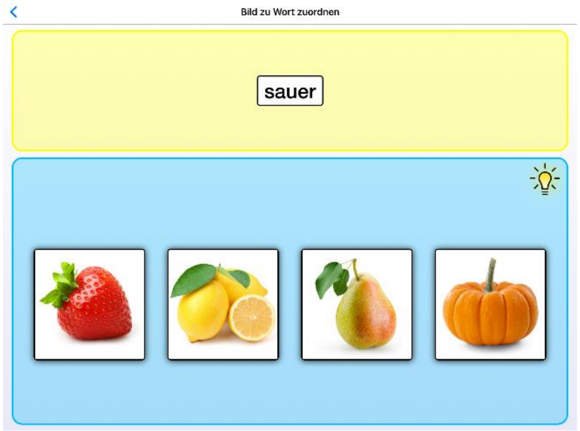

Figure 3: Exercise single word-picture matching

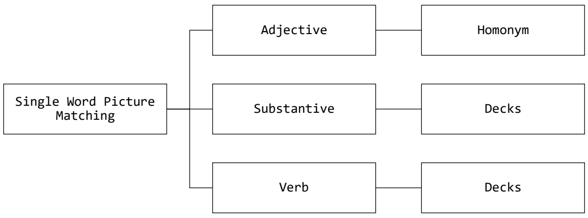

Figure 4: Exercise structure single word-picture matching

1.3 Multiple matching

The task of multiple matching is to match each object to another object. There are three different types of objects (word, picture, and image) and three corresponding different matching tasks, i.e. word-picture, picture-picture, and word-word matching. An example of word-to-word and picture-to-picture matching is shown in figure 5. To adapt the task to the patients, the exercise structure (figure 6) is hierarchically structured into decks focused on homonyms and antonyms.

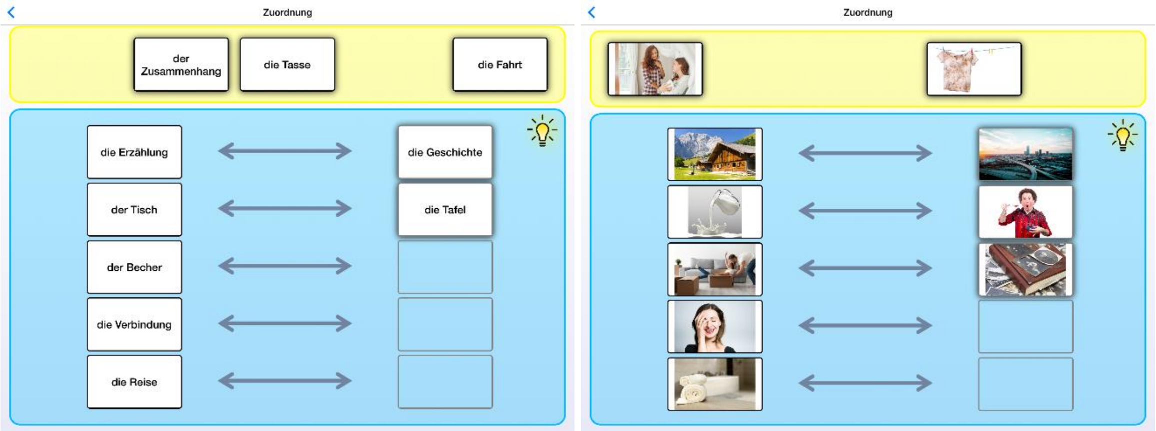

Figure 5: Left image: exercise word-to-word matching, Right image: picture-to-picture matching

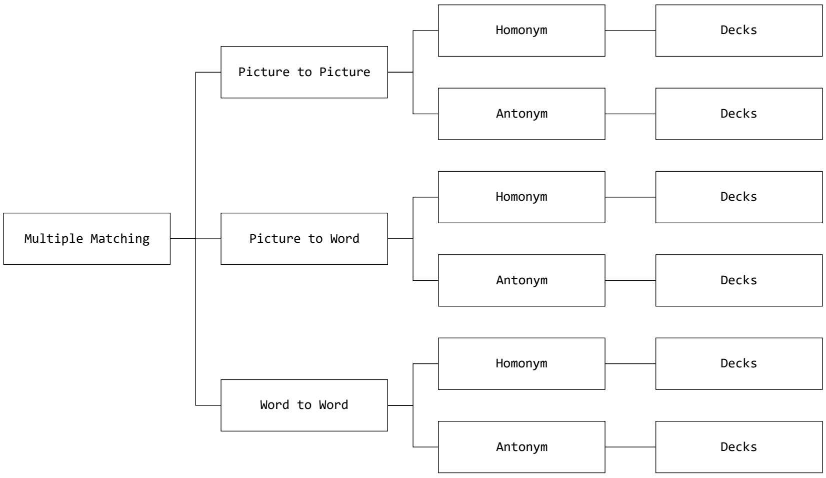

Figure 6: Exercise structure multiple matching

2 Category Insertion

2.1 *Word completion*

The task of word completion is to select the correct letter(s) and insert them into the correct position(s) of the word. For example (figure 7), the word we are looking for is **kalt** (cold). Thus, the correct letter to complete the word is the **a,** and **i** would be the distractor. The number of distractors can be varied. Additionally, as supportive elements to the picture, a video or audio file of a speech and language therapist spelling the correct word can be displayed. To adapt the task to the patients, the exercise structure (figure 8) is hierarchically structured into decks focused on phonematic criteria.

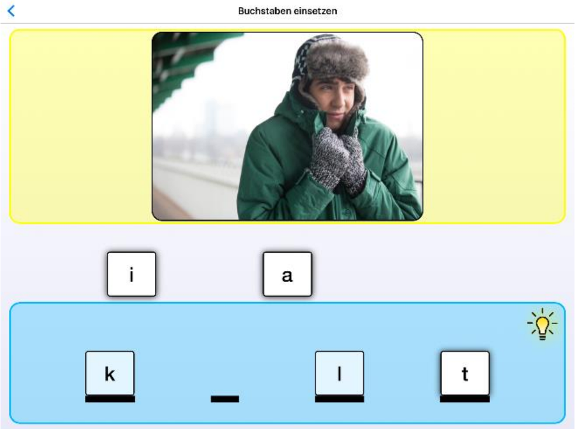

Figure 7: Exercise word completion

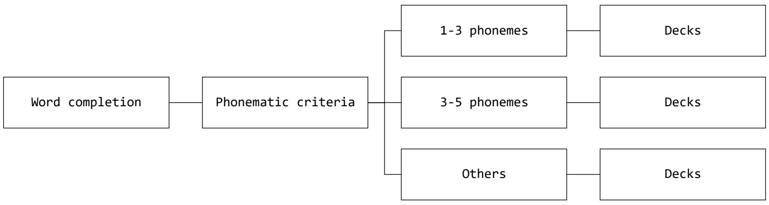

Figure 8: Exercise structure word completion

2.2 *Sentence completion*

The task of sentence completion is to select the correct word(s) and insert them into the correct position(s) to complete a meaningful sentence. For example (figure 9), the word we are looking for to complete the sentence is **kalt** (cold). Brandheiss (hot) would be the distractor. The number of distractors and the words to select can be varied. Additionally, as supportive elements to the picture, a video or audio file of a speech and language therapist spelling the correct word can be displayed. To adapt the task to the patients, the exercise structure (figure 10) is hierarchically structured into decks focused on semantic processing and grammatical syntactic processing.

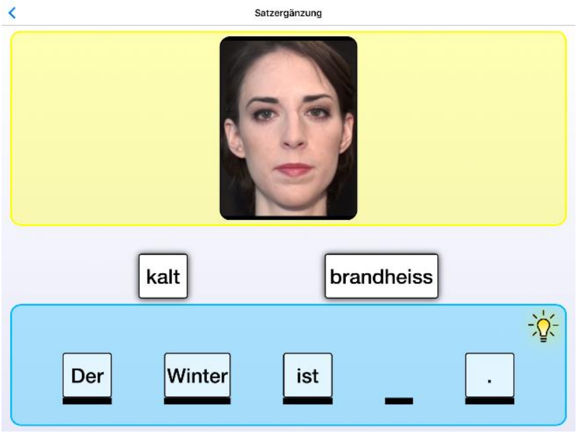

Figure 9: Exercise sentence completion

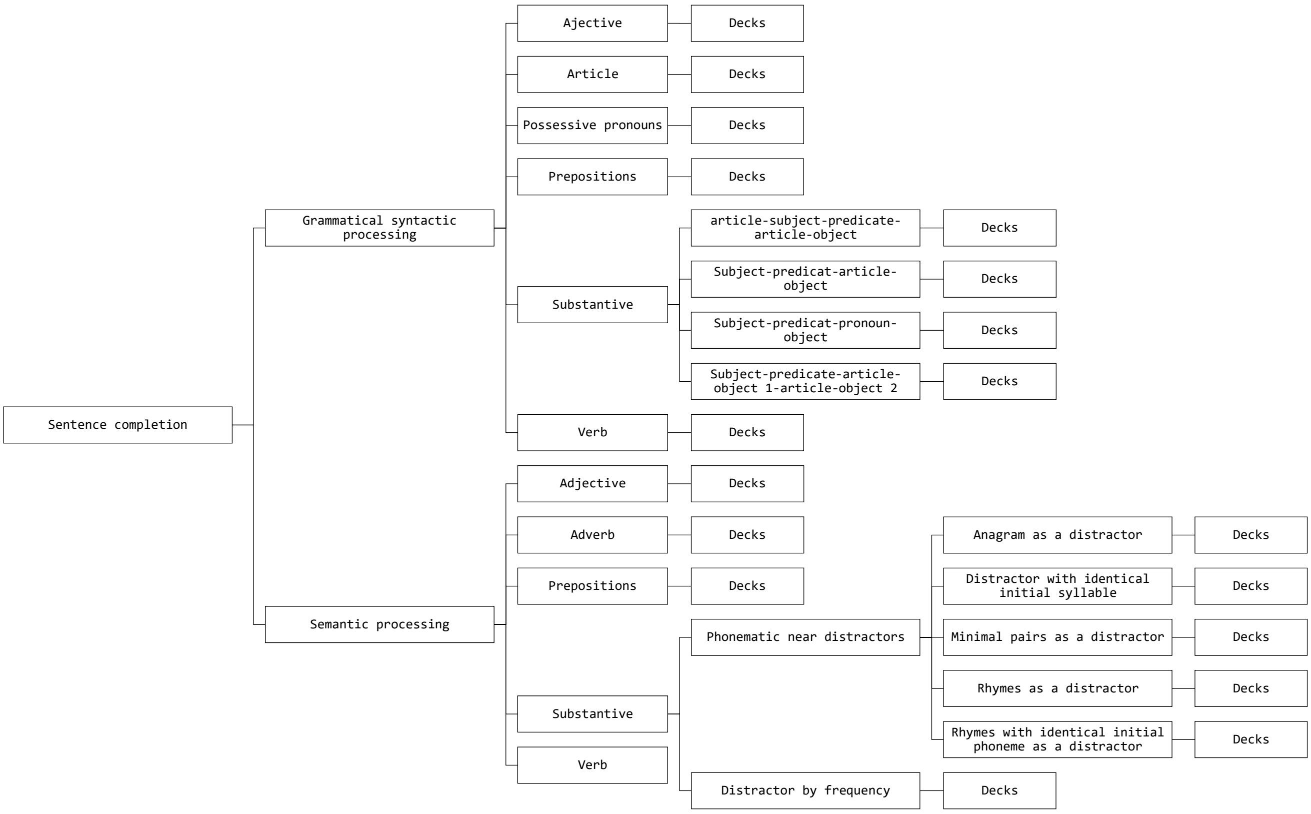

Figure 10: Exercise structure sentence completion

3 Category Sort

3.1 Anagram

The task of anagram is to bring the letters into the correct order to complete the word. For example (figure 11), the letters should be ordered to complete the word **Maler**. Additionally, as supportive elements to the picture, a video or audio file of a speech and language therapist spelling the correct word can be displayed. To adapt the task to the patients, the exercise structure (figure 12) is hierarchically structured into decks focused on phonematic and semantic criteria.

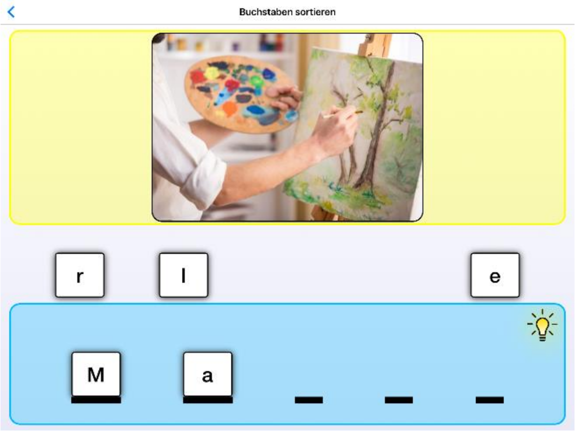

Figure 11: Exercise anagram

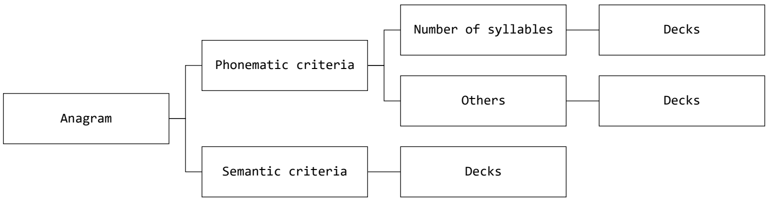

Figure 12: Exercise structure anagram

3.2 Sentence ordering

The task of sentence ordering is to bring the words into the correct order. For example (figure 13), the words should be ordered to complete the sentence “Der Wäschekorb ist nicht leer.” (The laundry basket is not empty.) Additionally, as supportive elements to the word, a video or audio file of a speech and language therapist spelling the correct word can be displayed. To adapt the task to the patients, the exercise structure (figure 14) is hierarchically structured into decks focused on sentences with three - six words.

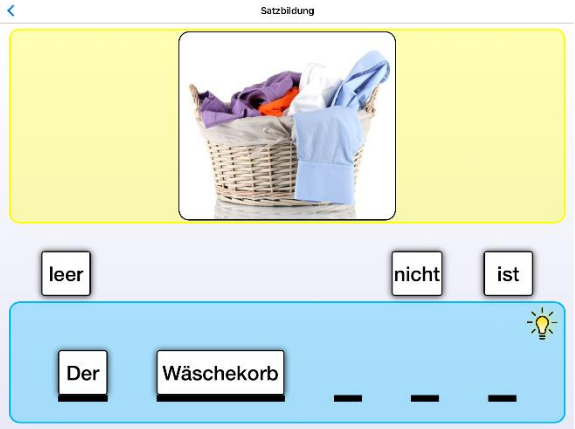

Figure 13: Exercise sentence ordering

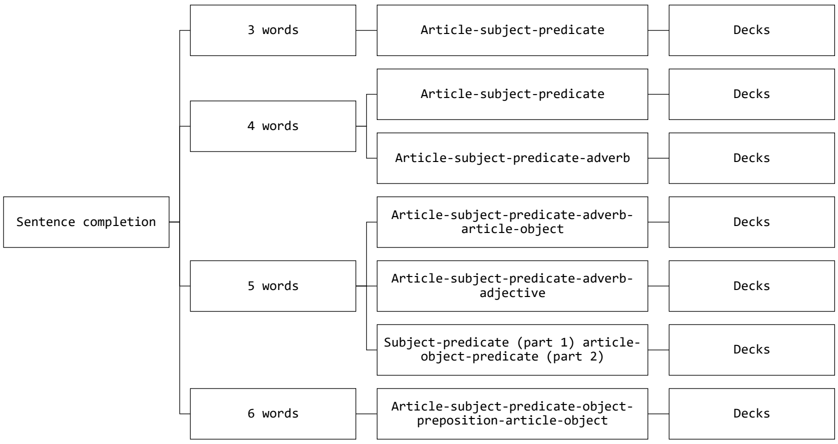

Figure 14: Exercise structure sentence ordering

4 Category Mimic

4.1 Word repetition

The task of word repetition is to repeat the audio-visually recorded spoken word by a speech and language therapist. For example (figure 15), the patient must mimic the word **dollar**. To adapt the task to the patients, the exercise structure (figure 16) is hierarchically structured into decks focused on sentences’ different dialects.

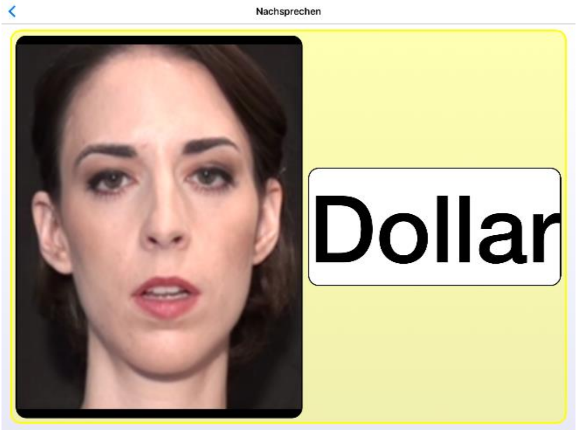

Figure 15: Exercise word repetition

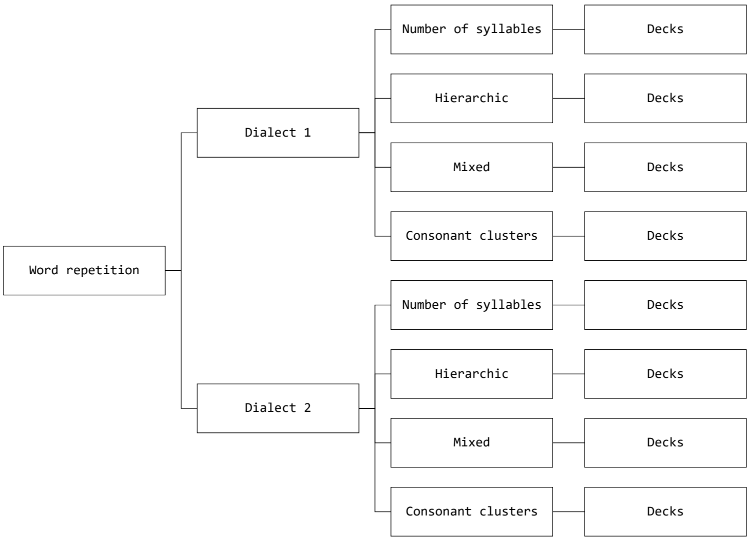

Figure 16: Exercise structure word repetition

5 Category Writing

5.1 Copy and recall

The task of copy and recall is to write or type the word presented. For example (figure 17), the word **Takt** must be copied and written correctly. To adapt the task to the patients, the exercise structure (figure 17) is hierarchically structured into decks focused on high- and low-frequency words.

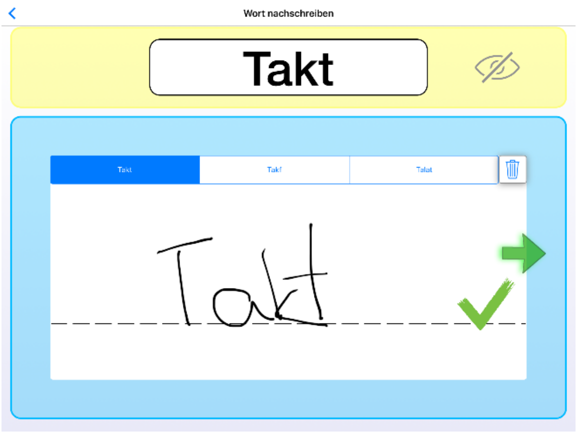

Figure 17: Exercise copy and recall

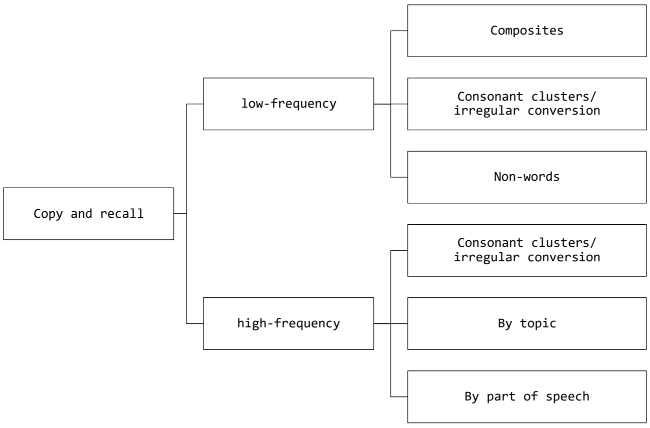

Figure 18: Exercise structure copy and recall

6 Category Comprehension

The task of comprehension is to select the correct answer to a question about the auditory, audio-visual, text-based respective image-based information. For example (figure 19), based on the image information the question is what the women carries with her for sure. The correct is answer is Pass, whereas the Paar (couple) and Fass (barel) are distractors. The number of distractors can be varied. To adapt the task to the patients, the exercise structure (figure 20) is hierarchically structured into decks focused on the four language modalities.

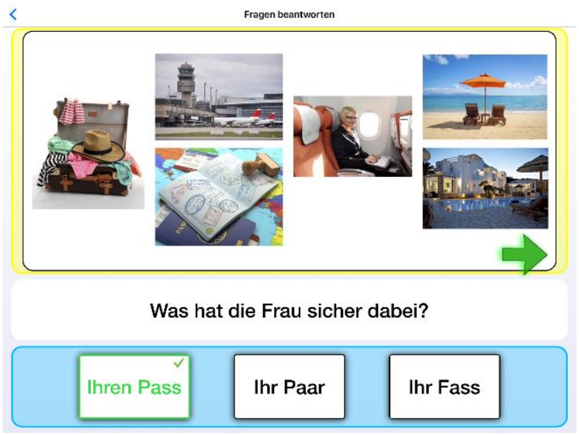

Figure 19: Exercise visual comprehension

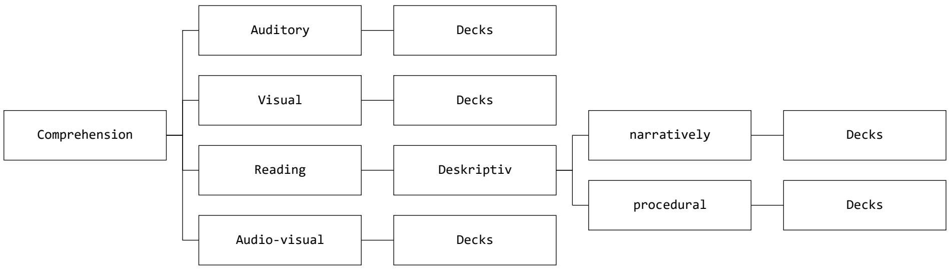

Figure 20: Exercise structure comprehension
